# An Upcycling Approach from Fruit Processing By-Products: Flour for Use in Food Products

**DOI:** 10.3390/foods14020153

**Published:** 2025-01-07

**Authors:** Laís Benvenutti, Fernanda Moreira Moura, Gabriela Zanghelini, Cristina Barrera, Lucía Seguí, Acácio Antonio Ferreira Zielinski

**Affiliations:** 1 Department of Chemical Engineering and Food Engineering, Federal University of Santa Catarina, Florianópolis 88040-900, SC, Brazil; lais.benvenutti@posgrad.ufsc.br (L.B.); fernandammoura@outlook.com (F.M.M.); gabriela.zanghelini@posgrad.ufsc.br (G.Z.); 2 Instituto de Ingeniería de Alimentos—FoodUPV, Universitat Politècnica de València, 46022 Valencia, Spain; mcbarpu@tal.upv.es (C.B.); lusegil@upvnet.upv.es (L.S.)

**Keywords:** flour, upcycling, fruit by-product, food product, residue, food sustainability, food innovation

## Abstract

The growing global population has led to increased food consumption and a significant amount of food waste, including the non-consumed parts of fruits (e.g., stems, rinds, peels, seeds). Despite their nutrient richness, these by-products are often discarded. With the rising interest in nutrient-dense foods for health benefits, fruit by-products have potential as nutritious ingredients. Upcycling, which repurposes waste materials, is one solution. White flour, which is common in food products like bread and pasta, has good functional properties but poor nutritional value. This can be enhanced by blending white flour with fruit by-product flours, creating functional, nutrient-rich mixtures. This review explores using flours from common Brazilian fruit by-products (e.g., jaboticaba, avocado, guava, mango, banana, jackfruit, orange, pineapple, and passion fruit) and their nutritional, physical–chemical properties, quality and safety, and applications. Partially replacing wheat flour with fruit flour improves its nutritional value, increasing the amount of fiber, protein, and carbohydrates present in it. However, higher substitution levels can alter color and flavor, impacting the sensory appeal and acceptability. While studies showed the potential of fruit by-product flours in food formulation, there is limited research on their long-term health impacts.

## 1. Introduction

Food loss and waste are escalating at alarming rates, manifesting in various forms throughout the production chain. Approximately one-third of the edible portions of food that were intended for human consumption is lost or wasted on a global scale, amounting to around 1.3 billion tons per year [1]. Regarding fruits and vegetables, 40% of produce is lost in the supply chain between harvest and consumption. In Latin America alone, this loss amounts to 50% of fruits and vegetables, highlighting the need to reassess food management practices [1,2].

Although “food loss” and “food waste” are often used as interchangeable terms, each reflects a slightly different aspect of poorly functioning food systems. According to the definitions set forth by the United Nations, “food waste” is all the food and the inedible parts associated with it that are removed from the human food supply chain, while “food loss” refers to all quantities of crops and livestock that are suitable for human consumption but are completely discarded or destroyed after harvest or slaughter, without being repurposed for any other use before reaching the retail level [3]. Food is lost or wasted at all stages of food manufacturing. Approximately 14% of global food production is lost from harvesting to retail alone, and at least another 19% at retail, food service, and consumption, although the latter is likely higher due to the limited availability of these data [3,4]. In developed countries, losses are prone to occur due to excess production or discarded fruits and vegetables that fail to meet the high commercialization standards. In developing countries, on the other hand, losses are often a consequence of the inadequacy of processing technology for these perishable raw materials. Even when harvesting and post-harvest practices are adequate, significant amounts of food waste are produced as a result of food processing, in which inedible parts, such as husks, seeds, peels, or stems, are discarded due to a lack of adequate technology to process them or rejection from consumers, or for being unsuitable for consumption [1,3]. By-products are defined as a substance or object resulting from a production process whose primary aim is not the production of that item. In other words, by-products are secondary flows of industries that can be economically reused [5].

In recent years, there has been a growing interest in food products that have high nutritional value and promote benefits to human health [6]. However, shifting towards sustainable production practices that involve reusing industrial waste and adding value to new products remains a challenge for the food industry. One way to improve the sustainability of food production chains is upcycling, which is the combination of the terms “up” and “recycling” [7]. The upcycling concept involves generating new uses for raw materials or food parts that would otherwise be discarded at the beginning of the production chain. Some target applications of upcycled products include foods, cosmetics, and biofuels [5,8].

Despite the current progress in reusing food by-products, their utilization poses numerous challenges [7,9], including (i) material availability, homogeneity, and stability; (ii) the selection of favorable methods for separating, purifying, and modifying different chemical materials; (iii) spoilage prevention before processing; (iv) ensuring the absence of toxic or pathogenic substances, such as microbial toxins, pesticides, heavy metals and other contaminants, when used for human or animal food; (v) the development of sustainable processing steps to create products with high-added value; and (vi) technological difficulties for large-scale production.

Regarding fruit wastes and by-products (FWB), a major challenge is the lack of specific legislation that regulates the suitability and safety of their inclusion in new products and food formulations [9]. Fruit by-products are liable to contain potential pathogens, hydrophobic organic contaminants [10], fungicides and pesticides [11], mycotoxins [12], metal contaminants (i.e., lead, mercury, arsenic, and cadmium) [13], and biogenic amines [13]. Therefore, FWB must first undergo a decontamination process to be considered adequate for application in the food industry. For instance, carcinogenic mycotoxins can be successfully removed from orange peels by absorption using hybrid bentonite [12]. Pasteurization and membrane processes, such as microfiltration, ultrafiltration, and nanofiltration, are also suitable alternatives to remove contaminants from plant extracts [13].

Among the various forms of reusing fruit by-products is their processing into flour, which is more shelf-stable and can be utilized in a wide variety of food products. While there are other alternatives for revalorizing fruit by-products (e.g., composting, bioethanol production, and the extraction of bioactive compounds), the production of flours or powders has the advantage to fully valorize these wastes or by-products for food applications, in addition to being rich in fiber, minerals, vitamins, and antioxidants [14]. Many studies have demonstrated the feasibility of partially replacing wheat flour with flour obtained from fruit processing by-products in various food products, such as bread [15], cookies [16], noodles [17], cakes [18], and others. Additionally, the by-product flour can be suitable for gluten-sensitive individuals, people with celiac disease, those who have dietary restrictions, or consumers who are reducing gluten consumption due to a “health halo” effect, in which gluten-free foods are regarded by consumers as healthier, even if it is not necessarily true [19].

The main unit operation in flour production is drying, a method that has become an alternative for conservation and for utilizing the nutrients present in fruits, minimizing waste. The process involves obtaining and selecting the raw material (i.e., fruit skins, seeds, and stems), followed by cleaning/sanitization and peeling or cutting to separate what will be used in the flour from the inedible parts. The resulting material is crushed and dried in an oven, in lyophilization tray dryers, under vacuum, or sun-dried [20]. After drying, a final milling step is required to obtain the powder with a specific particle size, which will partly determine its stability during storage, as well as its ability to interact with water and oil and to release bioactive compounds during the digestion process [21].

**Figure 1:**
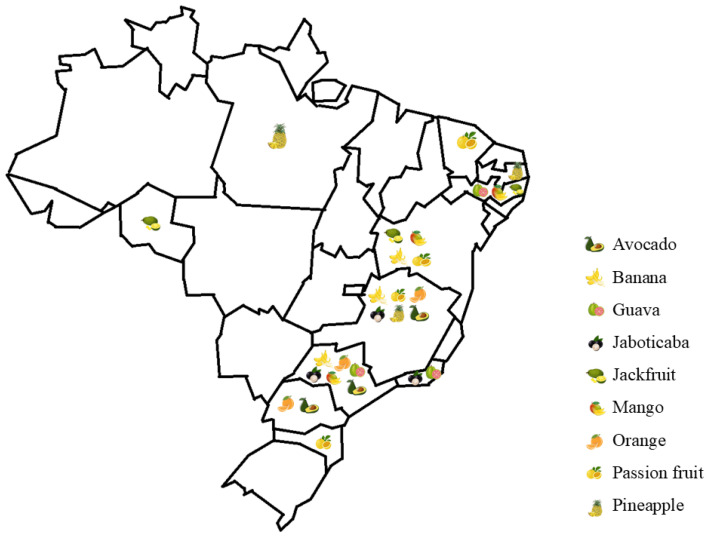
The main fruit by-products in Brazil, and the states in which each fruit is produced the most.

Therefore, this review explores the production of flour from fruit by-products of fruits that are vastly found in Brazil (Figure 1) (i.e., jaboticaba, avocado, guava, mango, banana, jackfruit, orange, pineapple, and passion fruit) and their potential use as ingredients in food applications that are based on the upcycling concept and have the potential to help reduce the environmental impact of food waste, add value to food by-products and meet consumer demands for differentiated and more sustainable products.

## 2. Bibliometric Analysis

**Figure 2:**
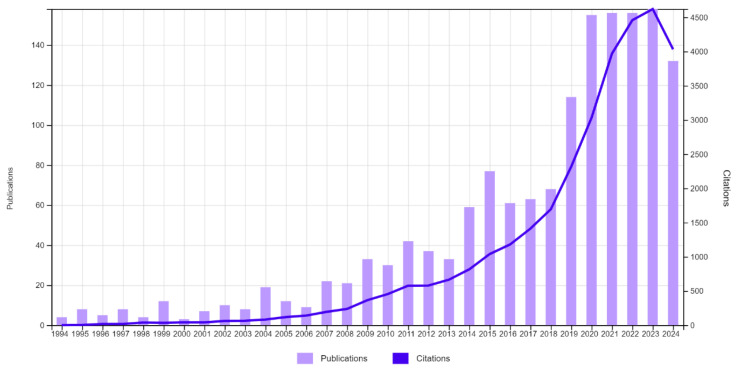
Number of publications on fruit waste flour and citations per year from 1994 to 2024. Source: Web of Science (2024).

For the bibliometric analysis, data were collected from the SCOPUS (scopus.com) and Web of Science (webofscience.com) databases using the keywords “flour” and the common and scientific names of the fruits. In total, 1872 documents were retrieved using SCOPUS, mainly research articles (83%), conference proceedings (9%), and reviews (3%), and 1526 were retrieved from Web of Science, 95% of which were research articles. The evolution of the number of publications and citations regarding fruit flours over the years highlights a growing interest in the topic, particularly in the last three decades, which concentrated 97% of the publications (Figure 2). The existing scientific production of fruit flour is mostly concentrated in Brazil (21.5% of the articles), India (12.5%), Mexico (8.1%), the USA (7.6%), and Nigeria (6.3%).

The data collected from SCOPUS and WoS were then filtered using the open-source bibliometric software, Biblioshiny 4.1, from RStudio (version 4.4.2). The selected documents were only those of the research and review article type, published in English, Portuguese, or Spanish, from 2004 to 2024, and with at least five citations per year. From these, 134 documents were carefully read and selected to extract information for writing the review.

## 3. Potential Use of Flours from Fruit By-Products Vastly Found in Brazil as Ingredients in Food Formulations

### 3.1. Jaboticaba

The jaboticaba is a fruit originally from the central region of Brazil. The fruit is characterized by a round shape, a bark with a dark purple color, and a white pulp containing a few seeds [8]. One peculiar aspect of this fruit is that it grows in clusters directly on the trunk of the jaboticaba tree, as presented by Benvenutti et al. [8]. The tree is native to a subtropical climate and can adapt to tropical climates, which means it can be cultivated in all regions of Brazil [22].

**Figure 3:**
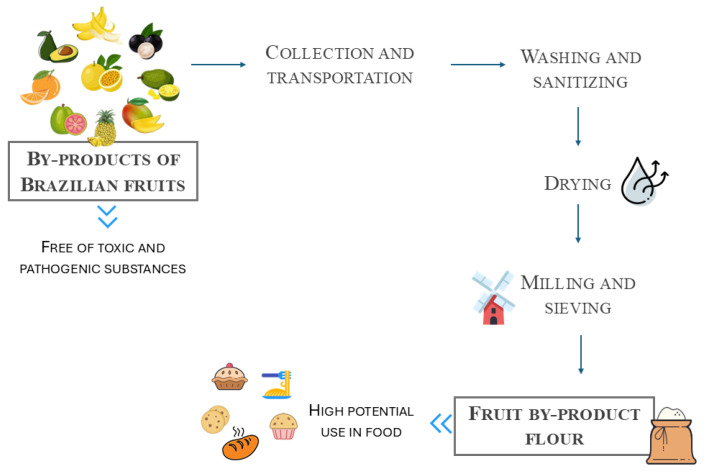
Diagram summarizing the process of obtaining flour from Brazilian fruit by-products as an alternative to add value to this underused material.

The industrial process of this fruit consists of selection, washing, sanitization, heating to extract the pigment from the peel, and the pulping of the fruit, which simultaneously separates the pulp from its by-products (seeds, peel, and residual pulp) [8] (Figure 3). Jaboticaba by-products comprise about 30% of its mass and are rich in fibers, protein, vitamins, minerals, and bioactive compounds [23]. Among the parts of by-products, the peel stands out for its higher concentration of fibers (33%) and phenolic compounds (2252 mg/100g) than that in seeds and residual pulp [8]. The main bioactive compounds present in jaboticaba fruit and its by-products are the phenolic compounds, which are mainly anthocyanin, followed by gallotannins, quercetin, and gallic acid [8,23]. Anthocyanins, mainly cyanidin-3-glucoside and delphinidin-3-glucoside, are present in jaboticaba fruit peel, from the ripening of the fruit. These compounds are responsible for fruit pigmentation and are correlated with potential bioactive properties, such as antioxidant, anti-glycemic, anti-inflammatory, and antiproliferative [8]. Gallotannins, e.g., castalagin and vescalagin, are found mainly in jaboticaba seeds and are related to antimicrobial activity and the inhibition of digestive enzymes, among other effects [24].

Table 1 summarizes the relevant data from studies on jaboticaba residue flour in food formulation. The flour from jaboticaba peels can be obtained after the steps of washing, sanitization, separation of the peel from the pulp and seeds, drying in a stove with air circulation at 60 °C, milling, and sieving (average particle size of 0.5 mm). The jaboticaba peel flour (JPF) was employed to replace wheat flour in different muffin formulations with 0%, 4.5%, 9%, 13.5% and 18% substitution. [25]. The most accepted formulation was with the addition of 9% of JPF, which presented a higher moisture content (7.86%), ash (1.40%), and dietary fiber (≥6%), besides presenting lower lipid (1.43%) and protein (1.50%) levels than the standard formulation (no addition of husk flour). When more than 9% of JPF was included in the formulation, the color of the muffins turned dark due to the large amount of anthocyanins present in the jaboticaba bark, hampering consumer acceptability. High levels of flour substitution with JPF also affected the product texture, as muffins with 13.5% or higher of JPF presented increased firmness. According to Walker et al. (2014) [26], a higher dough firmness is associated with fruits with high fiber content, as is the case of jaboticaba, since the fiber is hygroscopic and promotes greater water retention. Another factor that could increase dough hardness is the lower levels of wheat flour, which limits dough elasticity due to the lower presence of gluten. Despite this, low levels of JPF inclusion in doughs can effectively improve the nutritional profile with little impact on sensory characteristics.

JPF was also reported to be an effective ingredient for developing potentially functional pan bread. The addition of JPF (from 5 to 10% in formulation) reduced the total amount of carbohydrates, lipids, and protein in the final product, besides increasing the moisture, crude fiber, and antioxidant activity, compared to the control. The ingredient was also used to nutritionally enrich products such as cookies and cereal bars (Table 1), demonstrating the feasibility of using jaboticaba by-products to nutritionally enrich bakery products.

**Table 1:** Jaboticaba peel flour is used as an ingredient in food products.

Formulated Product	By-Product Type	Amount of Added Flour (%)	Amount Suggested (%)	Fortified Nutrients	Reference
Muffins	Peel	Substitution of 4.5, 9, 13.5, and 18% of wheat flour	9	Increased ash, moisture, and dietary fiber	Micheletti et al. [25]
Bread	Peel	Addition of 0, 5, 10 and 15% in formulation	5 and 10	Increase in moisture, minerals, and fiber	Ferreira et al. [27]
Bread	Peel and seed	Substitution of 10% of wheat flour	10	Increased fiber content	Faller et al. [28]
Cereal bar	Peel (mixed with soy okara)	Addition of 2.7, 5.4 and 8.1% in formulation	2.7	Increased protein and fiber content	Appelt et al. [29]
Cookies	Peel	Substitution of 30, 45 and 60% of total flour	0.3	Increase in fiber content	Zago et al. [30]

### 3.2. Avocado

The avocado (*Persea americana*) is a tropical and subtropical fruit that is native to Central America, which accounts for about a third of the 8.9 million tons of the fruit produced globally each year (FAOSTAT, 2022). It has a rough, dark green crust that covers a yellowish-green pulp that is rich in oils and is appreciated for its sensory and nutritional characteristics [31]. The fruit has been recognized for its health benefits, especially due to the presence of compounds such as omega fatty acids, phytosterols, tocopherols, and squalene, which are present in the lipid fraction [32]. While the high content of vitamins E, C, B6, β-carotene, and potassium in avocado pulp make it interesting for direct consumption in the human diet [33], avocado seeds also present a good nutritional profile, as they are rich in polyphenols (e.g., flavonoids), minerals, such as potassium and magnesium, and vitamins C, E, and K [34]. In addition, avocado pulp oil, which is rich in monounsaturated fat acids, including omega 9, can be recovered by mechanical or solvent extraction and applied in food, cosmetic, and therapeutic formulations.

Avocado seeds are the fruit’s main by-product, with approximately 148,000 t of seeds being discarded worldwide every year [31]. Another by-product is avocado pulp cake (about 25% of pulp mass), which is a fibrous material generated by avocado oil manufacturing [35,36]. Several studies have focused on upcycling avocado flour from seeds and pulp cake to be used in the food industry. Table 2 summarizes some relevant data on studies focused on avocado residue flour applied to food products.

In general, flour from avocado seeds or pulp cake is obtained after processes such as drying, milling, and sieving [36,37] (Figure 3). The literature reported a high moisture value of avocado seed flour, which is characterized by a high hygroscopicity (27.55%) [37]. The flour also presents a high lipid content, which results in high acidity and a high fiber content, a positive factor for the functionality of flour [36]. In a study by da Silva et al. (2019), avocado seed flour was applied by substituting 5, 10, or 20% of wheat flour in cookie formulations [37]. From the sensory analysis (70 panelists), the authors concluded that the better-accepted formulations were those with 5% avocado seed flour. In a similar study, Chaves et al. (2013) evaluated the substitution of 10% to 50% of wheat flour in cookies and concluded that the lower substitution degree presented better sensorial acceptance [36]. Olaleye et al. (2020) [38] proposed to apply avocado seed flour in weaning food to improve the nutritional quality of the formulation. The authors evaluated the addition of 10, 15, and 20% and concluded that blends with 20% avocado seed flour, 65% maize flour and 15% Irish potato flour showed the best combination in terms of nutritional and functional properties. These studies highlight that avocado seed flour presents a potential use in food formulations with a high nutritional value.

Besides improving the nutritional value of food formulations, avocado seed flour presents anti-proliferative, antioxidant, anti-inflammatory, and antimicrobial effects, suggesting its potential use as a functional ingredient. In addition, avocado seed flour can be an alternative ingredient for developing formulations for patients with diabetes mellitus, owing to its low sugar content associated with a high content of flavonoids, which contribute to the reduction in blood glucose levels [39].

**Table 2:** Avocado flour is used as an ingredient in food products.

Formulated Product	By-Product Type	Amount of Flour Added (%)	Amount Suggested (%)	Fortified Nutrients	Reference
Cookie	Seed	Substitution of 5, 10 and 20% of wheat flour	5	Increased moisture, ash, and carbohydrates	Silva et al. [37]
Cookie	Pulp cake from oil extraction	Substitution of 10 and 50% of wheat flour	10	Increase in fiber, ash, and carbohydrates	Chaves et al. [36]
Weaning foods	Seed	10, 15 and 20% in formulation	15	Increase in fiber, ash, and carbohydrates	Olaleye et al. [38]

These studies show that avocado flours have the potential for application in food products, although the food safety of the seed and pulp flour still needs to be further investigated.

### 3.3. Guava

The guava (*Psidium guajava*) is a tropical fruit produced on a large scale in Brazil, which is considered one of the main producers of guava in the world, with 582,832 t produced in 2023 alone [40]. The fruit is often described as a “Super Fruit” owing to its high levels of antioxidant compounds, such as anthocyanins and polyphenols [41], in addition to being a good source of vitamins A and C, phosphorus, iron, calcium, and dietary fiber [42].

Guava is either consumed fresh or processed into a wide variety of commercial products, such as pulp, juice, jam, or canned syrup, most of which require removing its seeds. It is estimated that 55% of the total production (233.368 t) is destined for processing, which generates an amount of waste (i.e., barks and seeds) equivalent to roughly 30% of the raw material (70.000 t) [43].

An alternative to using guava in its entirety in processed foods is by turning it into flour by drying and milling. Guava flour has been used to partially replace wheat flour in bakery products to nutritionally enrich them. Partially replacing wheat flour with up to 20% of guava flour in bread formulations has been shown to increase the amount of phenolic compounds by 135%, namely rutin, 2,4-dihydroxybenzoic, 3,4-dihydroxyphenylacetic and gallic acids, which are predominant in guava flour [44]. In addition to a higher phenolic content, bread enriched with guava flour presented a lower protein and starch content when compared to the control formulation (100% wheat flour), as well as an increased presence of volatile aroma compounds, such as α-humulene, β-caryophyllene, and limonene, which helped improve consumer acceptance, particularly in the 20% formulation [45].

Guava flour has also been tested in the manufacturing of cookies and biscuits. A partial replacement of up to 10% of flour content in cookies with flour from whole guava fruits allowed for the improvement in protein (6.54 to 9.03%) and crude fiber (0.24 to 1.8%) content while maintaining the moisture, fat, and ash levels. While up to 5% of wheat replacement improved the color, texture, taste, aroma, and overall acceptability of cookies, higher levels of substitution negatively impacted sensory acceptance [46]. Similarly, cookie formulations replacing wheat flour with up to 9% of guava seed meal improved the crude fiber content (from 0.52 to 3.65%) while maintaining the fat, ash, and protein levels. Although most organoleptic properties were unaffected by the addition of guava seed flour, substitutions above 6% slightly decreased the taste and texture perception [47]. In contrast, using guava peel flour to replace wheat flour in cookies increased the moisture and protein content. It also decreased the amount of fat and carbohydrates with increasing substitution levels and improved polyphenols and ß-carotene. Here, again, higher levels of substitution (70%) had a deleterious effect on cookie flavor and texture [48]. For all guava flours tested, a moderate substitution (5–6% for whole fruit or seed flour, and 50% for peel flour) seems to lead to the best balance between the nutritional and organoleptic properties in cookies.

A different application for guava peel flour is adding it to guava pulp juice to enhance its nutritional quality. While the addition of 1–5% guava peel flour allowed for the improvement in the content of dietary fiber, anthocyanins, and antioxidant activity, additions of 3% or higher also increased juice acidity, enhanced bitterness, and darkened its color, thus decreasing consumer acceptability [49].

In sum, guava residue flour can be used as a partial replacement for other flours and powders in bakery products and beverages, as summarized in Table 3. However, in both types of applications, high substitution levels risk hampering consumer acceptability due to deleterious effects on sensory properties.

**Table 3:** Guava peel flour is used as an ingredient in food products.

Formulated Product	By-Product Type	Wheat Flour Replacement (%)	Amount Suggested (%)	Fortified Nutrients	Reference
Bread	(whole fruit)	Substituting 10 and 20% of wheat flour	20	2- to-3-fold increase in phenolic compounds	Alves and Perrone [44]
Bread	(whole fruit)	Substituting 10 and 20% of wheat flour	20	Increase in phenolic compounds and antioxidants.	Castelo-Branco et al. [45]
Biscuits	(whole fruit)	Substituting 2.5, 5, 7.5 and 10% of wheat flour	5	Increase in protein and fiber content	Zafar et al. [46]
Cookie	Peel	Substituting 30, 50 and 70% of wheat flour	30	Increase in fiber, ash, polyphenols and β-carotene	Bertagnolli et al. [48]
Cookie	Seed	Addition of 3, 6 and 9%	6	Increase in fiber, ash and iron	El-Din et al. [47]
Juice	Pulp and peel	1, 3 and 5	1	Increase in phenolic compounds, antioxidants	Silva et al. [49]

### 3.4. Mango

The mango (*Mangifera indica* L.) is a fruit plant of the *Anacardiaceae* family that is widely cultivated and appreciated around the world, being the second most commercialized and fifth most produced tropical fruit worldwide, with 1.76 million t produced yearly in Brazil alone [40,50]. The fruit is roughly composed of the peel (9.80–14.30%), the pulp (66.10–72.40%), and the kernel (8.40–12.4%) [51]. Mango consumption and industrial applications are generally restricted to the pulp, whereas the peel and kernel are often discarded as waste despite their high content of fat, protein, crude fiber, and carbohydrates [18,50].

A mango seed consists of a hard outer shell encasing a starchy kernel, which, together, represent 9% of the total fruit mass and are rich in macronutrients and micronutrients (e.g., sodium, potassium, phosphorus, magnesium, and calcium), as well as flavonoids and phenolic compounds [50]. Another 15–20% of the fruit corresponds to the peel, which has a high content of dietary fiber, pectin, polyphenols, and carotenoids [52]. This good nutritional value highlights a good potential to repurpose the mango kernel and peel as flour to be incorporated into a variety of food applications, such as pasta and bakery products (Table 4).

Mango peel flour (MPF) has been used to partially replace wheat flour in the formulation of pasta as a means to improve its relatively poor nutritional value. Nur Azura et al. (2019) evaluated the partial replacement (up to 30%) of wheat flour with mango peel flour in yellow alkaline noodles. While a 30% substitution allowed for the significant improvement in moisture (by 1.2 times) and crude fiber content (by 15.2 times) and decrease in the amount of fat and carbohydrates (by 1.8 and 1.3 times, respectively), it also hampered gluten formation, resulting in pasta with a weaker structure and lower resistance to heat [53]. The high levels of substitution (30%) also impacted the sensory characteristics of the pasta formulation (i.e., taste, texture, color, and appearance), reducing overall consumer acceptance. One of the most notable changes with MPF addition was in color; pasta samples presented a darker coloration with increasing MPF concentration, which is attributed to the enzymatic oxidation of mango peel polyphenols by polyphenol oxidase. If wheat replacement with MPF is limited to 20%, most of these negative alterations can be avoided.

A similar study was conducted by Rudra et al. (2019) for fusilli pasta, this time using flour from mango kernel (MKF). The flours were first autoclaved to reduce their polyphenol content and thus, decrease their characteristic bitterness, then incorporated as partial substitutions (5–15%) for semolina in pasta formulations. The pasta incorporated with MKF presented lower water absorption, higher stickiness, and lower overall acceptability by consumers, which is likely a result of its darker coloration, particularly in the formulation with 15%. The results suggest that a relatively good sensory quality and consumer acceptance can be achieved with substitutions of up to 10% [54].

When compared to wheat flour, mango kernel flour has a higher bulk density and water absorption capacity and a lower oil absorption capacity and swelling index [18]. Such characteristics can be beneficial for their application as a partial replacement for wheat flour in bakery products such as cookies, cakes, and bread. When incorporated into composite cakes, MKF resulted in slightly lower levels of moisture and protein and higher fat content, in addition to decreasing its height and specific volume. It also significantly impacted the crumb and crust color, leading to a darker crust and crumb with increasing MKF concentration. While substitutions of 20% or lower were rated highly by consumers in terms of color, flavor, texture, taste, and overall acceptability, higher amounts had deleterious effects on flavor and texture, decreasing acceptability [18].

In bread doughs, substituting up to 25% of wheat flour with MKF slightly improved the protein, fat and crude fiber content (by 3.43%, 8.67%, and 29.73%, respectively), in addition to increasing the levels of phenolic compounds (by 25.57%), calcium, iron, zinc, and potassium (by 200%, 75%, 15%, and 14%, respectively) present. Despite improving the nutritional quality of bread, MKF inclusions significantly impacted (*p* < 0.05) its sensory aspects, particularly at substitutions of 20% or higher, which were characterized by markedly bitter tastes and darker colors that are likely a result of the higher presence of tannins [55]. Similarly, incorporating mango peel flour (MPF) in the formulation of a whole wheat bread improved its phenolic content (by up to 243% for a 5% substitution) but negatively affected bread texture, increasing moisture, hardness, and stickiness and decreasing porosity which, consequently, decreased bread height and volume. Increasing substitution levels of up to 5% also imparted a fruitier aroma and reduced the traditional bread aroma, in addition to darkening the crumb and crust color. These flavor and color changes are attributed to the additional sugar imparted by mango peels and the occurrence of non-enzymatic browning reactions during baking [56,57]. Nevertheless, up to 5% of MPF substitution was considered to result in bread with adequate volume and acceptable sensory characteristics [57].

When incorporated in biscuit doughs, mango peel flour improved nutritional properties by increasing the polyphenol and carotenoid content (by 8- and 14-fold, respectively), which led to a high antioxidant activity. It also improved the amount of dietary fiber, which, in turn, increased water absorption, as demonstrated by Ajila et al. (2008) [58]. At substitutions of 15–20% of wheat flour with MPF, the higher water content led to biscuits with increased hardness and smaller heights and diameters due to the formation of a more extensible gluten structure. As observed for bread and cakes, biscuits with MPF presented a darker coloration that is attributed to the high content of polyphenols and the presence of polyphenol oxidase and peroxidase in the mango peel. Although the high polyphenol content also imparted a bitter taste for biscuits with 15–20% MPP, substitution levels of up to 10% resulted in good consumer acceptability while maintaining a high antioxidant activity and sensory attributes comparable to the control [58].

In sum, both mango kernel and peel flours can be employed to increase the crude fiber content and antioxidant activity of bread, dough, and pasta, although higher addition levels may impart deleterious changes in color, texture, and flavor.

**Table 4:** Mango peel and kernel flours are used as ingredients in food products.

Formulated Product	By-Product Type	Wheat Flour Replacement (%)	Amount Suggested (%)	Fortified Nutrients	Reference
Biscuit	Peel	Substitution of 5, 7.5, 10, 15 and 20% of wheat flour	10	Increase in dietary fiber, polyphenols and carotenoids	Ajila et al. [58]
Composite cake	Peel	Substitution of 10, 20, 30 and 40% of wheat flour	20	Increase in fiber, ash and fat	Das et al. [18]
Fusilli pasta	Kernel	Substitution of 5, 10 and 15% of semolina	10	Increase in phenolic content and antioxidant activity	Rudra et al. [54]
Bread	Kernel	Substitution of 5, 10, 15, 20 and 25% of wheat flour	<20	Increased protein, dietary fiber, and phenolic compounds	Amin et al. [55]
Bread	Peel	Addition of 2.5, 5 and 7.5% to formulation	5	Increased polyphenols and carotenoids	Hasan et al. [57]
Bread	Peel	Addition of 1, 3 and 5% to formulation	3	Increased protein, dietary fiber, and phenolic compounds	Pathak et al. [56]

### 3.5. Banana

The banana is a fruit belonging to the *Musaceae* family, which is cultivated in tropical and subtropical regions around the world, constituting the staple food of many populations and being one of the most consumed fruits globally [59]. India is the largest producer in the world and Brazil is the fifth largest producer, with a production of about 6.8 million tons in an area of 457,910 thousand hectares in 2022 (FAO, 2024). Around 53% of Brazilian banana production is industrially processed, which generates a large amount of banana peel, the by-product that corresponds to about 40% of the total mass of fruit [59].

Banana peels consist of a massive amount of organic material to be managed. This material is widely used as organic fertilizer or animal feed. Currently, many studies are showing that the banana peel is rich in dietary fiber, phenolic compounds, and minerals such as potassium and magnesium [59]. Thus, banana peel flour has been proposed as a product with a long-shelf life that can be widely applied in food formulation. The preparation of banana peel flour (BPF) started with rinsing, followed by soaking in sodium metabisulfite, sodium hypochlorite, or citric acid solution to minimize enzymatic browning. After that, banana peels are sliced, dried, ground, sieved, and stored [59] (Figure 3). Table 5 presents some applications of BPF in food formulations, such as bread, cake, rissole, candies, and meat products.

**Table 5:** Banana peel powder is used as an ingredient in food products.

Formulated Product	By-Product Type	Amount of Flour Added (%)	Amount Suggested (%)	Fortified Nutrients	Reference
Gluten-free rissol	Peel	5 and 10% of rice flour substitution	5%	Increase in fiber content	Gomes et al. [60]
Flatbread baladay	Peel	5 and 10	10	Higher fiber content, protein, fat and ash	Eshak [61]
Candy	Peel	0, 5 and 10	0	Increase in fiber content	Oliveira Neto et al. [62]
Cake	Peel	5, 10 and 15% of wheat flour substitution	5	Higher humidity and total solids	Oliveira et al. [63]
Gluten-free biscuit	Pulp or peel	3.5 to 19% of pulp flour and 3.5 to 9.5 of peel flour	7.5% of pulp flour and 3.5% of peel flour	High levels of resistant starch, total phenolic compounds, total flavonoids and antioxidant activity	Leonel et al. [64]
Beef burger	Pulp or peel	3%	3% of green banana peel or pulp	Potential to substitute fat without hampering product quality	Bastos et al. [65]

In bread and cake formulation, banana peel flour was added by substituting other flours, such as wheat [61,63]. Eshak [61] substituted 5 and 10% of wheat flour for BPF in Egyptian bread formulation. The author concluded that bread formulations with BPF were acceptable for sensory assessment and contained more protein, fiber, and minerals than the control (100% wheat flour). However, to achieve acceptable nutritional and sensorial parameters, the bread can be prepared by replacing at most 10% of wheat flour with BPF. In addition, the bread with banana flour presented a higher water-holding capacity (WHC) and oil-holding capacity (OHC) than the control. Banana pulp flour (BF) and BPF were also applied in gluten-free cookie formulations [64]. The best formulation considering the physical and sensorial attributes was with the addition of 7.5% of BF and 3.5% of BPF, which showed higher levels of proteins, fibers, lipids, ash, total phenols, total flavonoids, and antioxidant activity than the control. However, the sensory results showed that increasing the percentage of banana peel flour resulted in greater changes in the color, aroma, and texture of cookies.

The flour of green banana pulp and peels was satisfactorily applied as fat substitutes in beef burger formulations [65]. The flour of the peels and pulp of green banana showed positive sensorial acceptance and higher water holding capacity (WHC) than the controls with and without added fat. A higher WHC allows for the enhancement of the juiciness and softness of beef hamburgers, increasing consumer acceptance.

### 3.6. Jackfruit

The jackfruit (*Artocarpus heterophylmlus*) is an Indian fruit that is cultivated in tropical regions worldwide and is known for its higher amount of protein (1.72%) when compared to other tropical fruits, such as the banana (1.09%) and mango (0.82%) [66,67]. The fruit presents easy cultivation and is widely distributed among all Brazilian territories, being more abundant in the coastal region [68]. The fruit consists of a thick outer rind surrounding several berries in which dark seeds are encased by a fibrous flesh (the pulp), in addition to a fibrous and stringy core. Ripe jackfruits weigh from 2 to 36 kg and can contain up to 500 seeds, which account for 8–15% of the fruit weight, 30–50% of which consists of pulp, 40–50% are rind and 5–10% is the core [69]. Jackfruits are generally consumed raw or after boiling, steaming, or roasting. Despite its high content of carbohydrates, proteins, minerals, and fat, jackfruits remain underutilized due to factors such as seasonality, logistical challenges, and preservation issues [70].

While jackfruit seeds are generally discarded as waste, they have gained attention, in the last few years, as a potential source of starch and protein [66] or as a substitute for cocoa, since their fermentation and roasting promote chocolate-like aromas [71]. Fermented jackfruit seed flour has been investigated as a substitute for cocoa powder in beverages such as hot chocolate [72] and cappuccino [69] due to its higher solubility and wettability and comparable water absorption capacity, apparent density and viscosity [73].

In addition to being used as a substitute for cocoa, jackfruit seed flour (JSF) has been exploited as a partial substitute for wheat flour in savory applications such as in pasta and bread, aiming to enhance their nutritional quality (Table 6). In the study performed by Abraham and Jayamuthunagai (2014) [74], the flour was obtained by drying seeds in an oven (60 °C, 6–7 h) and milling them into a powder, which was then incorporated in Rotini pasta to replace up to 20% of wheat flour. The addition of JSF to pasta increased paste viscosity for a 5% substitution, then decreased it again with increasing substitution levels, suggesting that the presence of JSF hampers starch resistance to temperature and shear stress. Overall, JSF additions improved the nutritional quality of pasta by increasing protein and ash contents, compared to pure wheat pasta, and preserving other nutrients and rheological characteristics. A 10% JSF substitution in pasta resulted in higher consumer acceptability in terms of flavor, mouthfeel, appearance, color, and overall quality, as higher levels tended to result in overly hard pasta [74].

Jackfruit rind is another by-product with a good potential for use in food applications given its high content of dietary fiber (~47%), particularly insoluble fiber, and high oil and water holding capacities (about five and seven times higher than wheat flour, respectively) [15]. Feili et al. (2018) incorporated jackfruit rind flour (JRF) in bread doughs to improve their nutritional properties [15]. The flours were obtained by rinsing the rinds in boiling aqueous solutions, followed by drying (50 °C for 24 h) and milling, then incorporated in bread formulations to replace up to 15% of wheat flour (Figure 3). The inclusion of increasing amounts of JRF in bread doughs increased the moisture, crude fat, and crude fiber contents while decreasing the amount of crude protein, carbohydrate, and calories. JRF addition also improved resistant starch, which could have a beneficial effect on digestion by acting as a dietary fiber [15]. However, inclusions above 5% JRF tended to reduce bread volume and increase its density, resulting in a harder, chewier, and less cohesive bread. While breads containing JRF had a darker coloration and were negatively affected in terms of taste, color, and softness, their overall acceptability was comparable to wheat bread when inclusions were limited to 5% JRF [75].

**Table 6:** Jackfruit seed and jackfruit peel flours are used as an ingredient in food products.

Formulated Product	By-Product Type	Amount of Flour Added (%)	Amount Suggested (%)	Fortified Nutrients	Reference
Cappuccino	Seed	Substitution of 50, 75 and 100% of cocoa powder	50	Higher wettability and solubility	Spada et al. [69]
Rotini pasta	Seed	Substitution of 5, 10, 15 and 20% of wheat flour	10	Increase in dough firmness	Abraham and Jayamuthunagai [74]
Bread	Rind	Substitution of 5, 10 and 15% of wheat flour	5	Fiber increase	Feili et al. [75]
Bread	Rind	Substitution of 5, 10 and 15% of wheat flour	5	Fiber increase	Feili et al. [15]

### 3.7. Orange

The orange is the most popular fruit consumed as a fresh fruit or as a drink/juice. Brazil is the largest orange producer in the world (16 million t), followed by China (7.7 million t), the European Union (5.8 million t), Mexico (4.8 million t), and the United States (3.1 million t) [76]. Oranges are mainly processed into juice, leaving about 20% of the fruit as peels as the processing by-products, in addition to seeds and albedo.

Owing to a high content of dietetic fiber, vitamins, minerals, and phenolic compounds, orange peel can result in a flour rich in antioxidant properties and with potential benefits for the digestive system [77]. Orange peel flour (OPF) is obtained after relatively simple steps such as washing, drying in a hot air oven, and milling (Figure 3). The peels must be stored at 4 °C, approximately, in a polyethylene packaging to avoid moisture absorption [77]. According to Galvan-Lima et al. (2021) [78], the greatest challenge for turning orange peel into flour is the moisture they present (about 75–90%). Thus, drying at low temperatures, specifically between 35 °C and 60 °C, is considered the most appropriate method to preserve the nutrients and prevent the growth and reproduction of microorganisms in the peels. This temperature range is critical to ensure minimal nutrient loss while effectively removing moisture, being the most appropriate method to preserve nutrients and prevent the growth of microorganisms. Furthermore, orange peels can contribute to organoleptic properties, such as aroma and flavor, as they contain limonene, a compound associated with citrusy aroma notes. Thus, orange peel flour can contribute to the flavoring of foods and preserves.

Obafaye and Omoba (2018) analyzed OPF at different wheat flour substitution levels (Table 7) in millet biscuit production [77]. Biscuits with 20% of OPF had increased amounts of ash, from 2.01 to 2.97%, crude fiber content from 0.25 to 0.54%, carbohydrates from 51.49 to 57.8%, and food energy values, from 466.29 to 507.95 kcal, in addition to the increase in potassium phosphorus levels. The inclusion of orange peel in the millet cracker also provided an increase in the contents of total phenolics (from 5.84 to 11.87 mg of gallic acid equivalents per gram), flavonoids (from 1.20 to 8.12 mg of quercetin equivalents per gram), and an increase in dietary fiber (from 4.84 to 5.27%). However, the moisture of the crackers decreased from 5.16 to 2.65%, fat decreased from 24.31 to 21.47%, and protein content decreased from 16.79 to 14.49%. The taste of the biscuit was also negatively affected by the addition of OPF due to the presence of limonene (a terpenoid that is associated with citrus flavor) in the orange peel.

A current technological challenge is to produce hamburgers that are low-fat, non-brittle, juicy, and flavorful. To tackle this challenge, Mousa [79] used polysaccharides (gum arabic, pectin, and sodium carboxymethyl cellulose) combined with peel flours from several citrus fruits (pomegranate, orange, and tangerine) to improve the quality characteristics and sensory attributes of a 95% fat-free hamburger. Hamburgers formulated with orange peel flour (3%) had a lower hardness value, which means a softer hamburger. In addition, a sensory analysis showed that the hedonic ratings for color, aroma, texture, flavor, and general acceptability did not differ from the control sample (without flour replacement). The addition of orange peel flour, together with tangerine and pomegranate peel flours, led to hamburgers with more red and yellowish colors due to the fruits’ natural pigments.

Ademosun, Odanye, and Oboh (2021) sought to produce low-glycemic antioxidant-rich pasta using wheat flour (WF), green banana flour (UPF), and powdered orange peel. The resulting product was tested in diabetic rats [17]. Different formulations were made with different percentages of UPF, WF and 10% orange peel powder (OPP). The highest total flavonoid content found was for the W55-UP35 formulation (55% WF, 35% UPF, and 10% OPP), which presented the highest increase in total phenolic content (from 22.04 to 7.62 mg of gallic acid equivalent per gram) and total flavonoid (from 0.57 to 3.38 mg of quercetin equivalents per gram). In addition, the consumption of the pasta led to higher glycemic risk values, decreased blood glucose, and higher phenolic content, especially flavonoids, in rats, when compared to the W55-UP35 formulation.

Cakes enriched with orange peel (OPF) and passion fruit peel (PFPF) flours were studied by Oliveira et al. (2016) [80] in different formulations: standard cake (wheat flour), wheat flour + 20% PFPF, wheat + 12.5% OPF and wheat flour +10% PFPF + 6.25% OPF. While the cakes with PFPF + OPF had the lowest protein values when compared to the standard cake, they also had higher levels of carbohydrates. In the sensory evaluation, the tasters preferred the cake with only wheat flour and orange peel flour in terms of appearance, texture, and flavor.

**Table 7:** Orange peel powder is used in fortifying food products.

Formulated Product	By-Product Type	Amount of Orange Flour Added (%)	Amount Suggested (%)	Fortified Nutrients	Reference
Pearl millet for biscuit	Peel	5, 10, 15 and 20%	5 and 10%	Increased fiber content, ash, carbohydrates, and antioxidant activity	Obafaye and Omoba [77]
Burger	Peel	3% and 2%	3%	Did not impair sensory attributes and protect the product against bacterial proliferation	Mousa [79]
Pasta	Peel	10% of wheat flour substitution	10	Improved phenolic content	Ademosun, Odanye and Oboh [17]
Cakes	Peel	12.5 and 6.25% of wheat flour substitution	12.5	Increase in dietary fiber content	Oliveira et al. [80]
Bread	Peel	3,6 and 9 of wheat flour substitution	3	Increase in ash content, fiber, carbohydrates, and phytochemicals levels	Okpala and Akpu [81]

Okpala and Akpu (2014) [81] produced bread enriched with orange peel flour (Table 7). With the addition of 3% of OPF, there was a reduction in protein (8.2–2.7%) and fat (0.8–1.7%); however, there was an increase in ash (2.3–4.3%), fiber (0.6–5.8%) and carbohydrates (59.9–62.1%) when compared to bread with wheat flour alone. As OPF has a large amount of fiber in its composition, it causes a decrease in moisture and in the amount of gluten, thus resulting in a decrease in the specific volume. (from 5.3 to 3.2 cm^3^/g). For most of the sensory characteristics studied, the samples did not differ significantly from the control sample. However, the bread with 3% of OPF presented deleterious effects on texture when compared to the control, and the bread with 9% of OPF presented lower ratings for all sensory attributes, being characterized by a higher bitterness and browning of the bread crust.

### 3.8. Pineapple

The pineapple (*Ananas comosus*) is one of the main tropical fruits commercialized worldwide, with a global production of 29.4 million tons in 2022 led by Indonesia, the Philippines, Costa Rica, and Brazil (FAOSTAT, 2022). While the fruit’s pulp is highly appreciated for its flavor and its high levels of minerals (e.g., manganese, magnesium, and potassium), vitamins (A, C, B1-B6, and B9), and phenolic compounds [82], it only constitutes about half of the fruit’s weight. The other half corresponds to by-products such as the peel (30%), stem (7%), and crown (13%), which are typically discarded during production and consumption [83]. Given the extent of pineapple production, this represents a massive amount of waste generated annually from pineapples alone.

While the pineapple crown is typically discarded by the food industry, it is a good source of lignocellulosic material, in addition to containing virtually as much protein as the pulp. In this sense, its potential use as an ingredient in food preparations has been tested by producing an oven-dried pineapple crown flour, which was characterized by a yellowish hue and contained approximately 9.3% humidity, 5.85% ash, 1.86% lipids, and a high fiber content (67.22%), primarily consisting of insoluble fiber (87% of the total fiber) [84]. Although this flour was not directly applied to food formulation, its high fiber content suggests it could potentially be used in dietary fiber supplements, functional foods, or as an ingredient to increase the fiber content in various food products.

Pineapple peel is another by-product whose high content of insoluble fiber suggests a good potential for application in low-calory and high-fiber foods. Pineapple peel flour (PPF) added to the formulation of cereal bars at concentrations of up to 6% has been shown to improve their moisture and fiber content while maintaining texture and sensory quality. Higher additions, however, reduced the moisture content and significantly altered the texture, decreasing overall consumer acceptance [85]. Pineapple peel flour has also been tested on bakery products such as muffins, biscuits, and bread (Table 8). In a study comparing gluten-free muffins in which 60% of rice flour was replaced with PPF alone or combined with banana peel flour and/or pumpkin seed flour, the formulations with PPF alone or combined with pumpkin seed flour led to the highest scores for flavor and overall acceptance, resulting in the greatest purchase intention. These formulations also had a higher content of insoluble fiber and total dietary fiber and a lower amount of lipids [86]. These results suggest that PPF could be included in gluten-free formulations without deleterious effects on sensory quality and consumer acceptance.

Yet another pineapple by-product is its core, which, when dried and milled into flour, contains about half the amount of fibers of PPF, while having a higher moisture and a higher content of carbohydrates and ashes [87]. De Toledo et al. (2017) [88] studied the effect of partially replacing wheat flour with pineapple core flour (PCF) in cookie formulations. When compared to the control (100% wheat flour), cookies containing 15% of PCF had a lower moisture and lipid content and higher levels of ash and insoluble fiber (1.05%) than the control sample without the by-product flour. While the inclusion of 15% of PCF resulted in darker-colored and thinner cookies, these differences were not perceived during consumption, as the 15% PCF cookies were rated similarly to the control in terms of appearance, texture, and overall impression. PCF cookies were also perceived as having a more pleasant taste than the control and had a higher acceptance rate and buying intention.

In addition to being a source of fiber, pineapple by-products have demonstrated good potential as prebiotic ingredients in preparations containing probiotic bacteria, such as Lactococci and Lactobacilli [89]. Sah et al. (2016) [90] investigated the impacts of adding pineapple peel powder (PPP) to yogurt formulations on its physical–chemical, sensory and rheological characteristics. The peels were first blanched for 30 min at 90 °C to inactivate proteolytic enzymes, then freeze-dried and ground into a powder, which was added to milk bases at 1% *w*/*v*. The addition of PPP decreased fermentation time by 25%, resulting in yogurts with lower firmness, lower apparent viscosity, and a yellowish coloration.

**Table 8:** Powdered pineapple by-products are used as ingredients in fortifying food products.

Formulated Product	By-Product Type	Amount of Flour Added (%)	Amount Suggested (%)	Fortified Nutrients	Reference
Cereal bar	Peel	Addition of 3, 6 and 9% to formulation	6	Increased crude fiber content	Damasceno et al. [85]
Cookies	Core	Replacement of 5, 10 and 15% wheat flour	15	Increased fiber	De Toledo et al. [88]
Muffins	Peel	Substitution of 13.3, 20 and 40% of rice flour	13.3 or 40	Increased fiber and improved overall acceptance	Brigagão et al. [86]
Yogurt	Peel	1	1	Decreased process time, improved fiber and decreased firmness.	Sah et al. [90]

### 3.9. Passion Fruit

The passion fruit is a tropical plant that belongs to the Passifloraceae family. Having more than 60 native species of passion fruit, Brazil stands out in the global market as the world’s largest producer of the fruit, with an annual production of over 700,000 t [40,80]. The most consumed species in the country is the yellow passion fruit (*Passiflora edulis* Sims), roughly 60% of which is consumed as fresh fruit. The other 40% produces juices and/or pulp, generating a large amount of by-products [80].

Passion fruit by-products include the peel and albedo (white inner part), which represent 50–60% of the fruit’s weight, and the seeds, which account for 4–12% of the fruit [16]. While these by-products are generally discarded, they are sources of carbohydrates, proteins, fibers, vitamins, and minerals that could be employed as ingredients in functional foods and applications, such as bakery products, yogurts, and confectionery.

Passion fruit albedo was crushed, dried (60 °C, 24 h) and milled into a powder [91] (Figure 3). Passion fruit albedo flour (PFAF) is characterized by a high fiber content (52.34% insoluble fiber and 19.45% soluble fiber) and a low protein (0.35%) and fat (1.00%) content, in addition to containing a considerable amount of vitamin C (377.36 mg/100 g) and pectin (40.5%), as well as multiple bioactive compounds, such as polyphenols (18.88 mg/100 g), flavonoids (13.51 mg/100 g), anthocyanins (1.74 mg/100 g) and chlorophyll b (69.65 mg/100 g) [92]. The high water holding capacity (13 g/g) and swelling capacity (37 g/g) of PFAF suggest that it could be applied as a thickener, as a foam and emulsion stabilizer, or in products that require hydration, such as bakery items and cooked meat products [91], as presented in Table 9.

PFAF has been combined with starfruit flour at proportions of 25, 50, and 75% and added to cereal bar formulations, along with rolled oats and rice flakes. While all of the formulations were highly rated in terms of aroma, flavor, and texture, the bars with only 25% of PFAF received the highest ratings for all of the attributes analyzed, especially color [93]. Another use for PFAF is as a partial replacement for wheat flour in cookie formulations, as described by Ning et al. (2020). While the incorporation of PFAF improved the contents of dietary fiber and polyphenols in the cookies, thus enhancing its antioxidant properties, substitution levels of above 6% resulted in a darker coloration, an increased hardness, and less pleasant flavor and aroma, significantly decreasing consumer acceptance. The starch digestibility of cookies also tended to decrease with increasing PFAF concentrations, likely due to the higher amount of fiber [16].

When incorporated in cakes as a partial replacement for wheat flour (20% *w*/*w*), PFAF allowed for the increase in fiber content by three-fold and slight decrease in the amount of carbohydrates. While the inclusion of 20% of PFAF had no significant impacts on cake appearance, it had deleterious effects on odor, flavor, and taste. An alternative that allowed for the better preservation of the cake’s sensory quality, while improving fiber content, was replacing 16.25% of wheat flour with a combination of PFAF (10%) and orange peel flour (6.25%), thus improving consumer acceptance [80]. In a similar study, the inclusion of a commercial yellow passion fruit peel flour (PFPF) in cake formulations to replace 7, 10 or 20% of wheat flour also increased the fiber content with increasing substitution levels (from 0.84 to 1.86%), resulting in a firmer texture. The addition of PFPF led to important changes in cake color, regardless of substitution level. While a 7% substitution had no deleterious effect on flavor, aroma, and texture when compared to the control (100% wheat flour), the 10% PFPF sample had the lowest ratings for flavor and texture and, consequently, the lowest consumer acceptance. Interestingly, higher substitution levels appear to reverse some of the deleterious effects in the sensory aspects, as the 14% PFPF cakes were comparable to the control and the 7% sample, in terms of perceived flavor, aroma, and texture. According to the authors, this could be related to the harder texture of the cakes with higher PFPF content, which might have affected the perception of other sensory attributes [94].

Passion fruit peels (epicarp + mesocarp) were dried in a greenhouse (90 °C, 11 h) and milled into flour that was incorporated in dietary cookies to replace 10, 20, or 30% of wheat flour. While PFPF increased the amount of ashes and dietary fiber content for all concentrations tested when compared to the control (100% wheat flour), it had no significant effects on the moisture, protein, lipid, and carbohydrate content. As observed previously in cakes, the sensory acceptance of the formulations with PFPF did not follow a linear pattern. While the formulations with 10 or 30% PFPF inclusion were rated similarly to the control for all sensory aspects, a 20% substitution had deleterious effects on appearance, flavor, and taste, resulting in the lowest global acceptance [95]. This was confirmed by Andrade et al. (2018), who observed that PFPF inclusions of up to 10% in cookies allowed for the improvement in fiber and ash content while having virtually little effect on sensory attributes and consumer acceptance [96].

Passion fruit flour can be a good alternative to replace wheat flour in gluten-free products. Ribeiro et al. (2018) prepared fresh gluten-free pasta in which rice and maize fours were partially replaced with PFPF (10 or 20%). The inclusion of PFPF increased water absorption with increasing concentrations and resulted in pasta with a darker coloration, thus decreasing consumer acceptability, particularly at 20% replacement. Despite being less appreciated by consumers than the control (no PFPF) in terms of taste, color, and overall appearance, the formulation with the addition of 10% PFPF received sensory scores that were close to the control and allowed for the increase in fiber and ash content [97].

The use of passion fruit by-product flours in food-related products can also extend to beverages, as demonstrated by de Toledo et al. (2018) for drinkable yogurts [98]. Flours from oven-dried (60 °C, 24 h) passion fruit peels and seeds (PFF) were added to passion fruit yogurt preparations at concentrations of 2 to 8% as sources of fiber and minerals. The addition of PFF increased the dietary fiber content by up to 17 times, with an insoluble to soluble fiber ratio ranging from 2:1 (0% PFF) to 3.1:1 (8% PFF), which helped delay syneresis during storage. When compared to the 0% formulation, PFF addition also increased the amount of minerals such as potassium, magnesium, and manganese. Despite the nutritional benefits, including PFF in the yogurt formulations resulted in a darker color, an enhanced bitterness, a grainier texture, and a higher viscosity with increasing PFF concentration, ultimately hampering consumer acceptance. Furthermore, the presence of PFF promoted yeast and mold growth over extended storage (above 14 days). Among all of the tested formulations, the yogurts with 2% of PFF presented the best compromise between nutritional benefits, physicochemical characteristics and consumer acceptance [98].

**Table 9:** Powdered passion fruit by-products are used as ingredients in fortifying food products.

Formulated Product	By-Product Type	Amount of Flour Added (%)	Amount Suggested (%)	Fortified Nutrients	Reference
Cookie	Peel	Substitution of 10, 20 and 30% of whole wheat flour	30	Increased fiber and ash	Garcia et al. [95]
Cookie	Albedo	Substitution of 3, 6 and 9% of wheat flour	6	Increase in fiber content, ash, and phenolic compounds	Ning et al. [16]
Stuffed cookie	Peel	Substitution of 5, 7.5 and 10% of wheat flour	10	High fiber content, protein, ash and lipids	Andrade et al. [96]
Cake	Albedo	Substitution of 10 and 20% of wheat flour	10	Increase in fiber content and decrease in carbohydrates.	Oliveira et al. [80]
Cake	Peel	Substitution of 7, 10 and 14% of wheat flour	14	Increased fiber content	Miranda et al. [94]
Gluten-free pasta	Peel	Substitution of 10 and 20% of rice and maize flour	10	Increased fiber content and ash	Ribeiro et al. [97]
Yogurt	Peel and seed	Addition of 2, 4, 6 and 8% to formulation	2	Increased fiber content, potassium, magnesium, and calcium	de Toledo et al. [98]
Cereal bar	Albedo	Addition of 3, 6 and 9% flour to formulation	6	Increased moisture and fiber	Bordim et al. [93]

## 4. Discussion

Fruit by-product flours have shown potential as substitutes for wheat flour in doughs and batters, enhancing their nutritional value by increasing dietary fiber, protein, and antioxidants, as well as vitamins and minerals [52,59,74,92]. Common fruit by-products include (1) rinds and peels, which are typically rich in fibers and phenolic compounds; (2) albedos, which are sources of pectin, fibers, and antioxidants; and (3) seeds, which contain considerable amounts of fiber, lipids, polyphenols and, often, lignocellulosic components. The obtention of flours from these by-products typically involves washing, sanitization, destemming, separating the peel from the pulp and seeds, drying (often at 50–60 °C), milling, and sieving to a specific particle size [20].

### 4.1. Techno-Functional Characteristics of Fruit By-Product Flours

The use of fruit by-product flours across different food categories is subject to their techno-functional properties, such as the following: water solubility, which indicates the amount of solute that can dissolve in a specific volume of water at a given temperature; emulsifying properties, which indicate the ability of a substance to stabilize a mixture of two immiscible liquids by reducing surface tension and preventing separation; foaming properties, which denote the ability to create stable foams when air is incorporated into a liquid; gelling properties, which refer to the ability of a substance to form gel-like structures when mixed with water; and water- and oil-holding capacities, which are the ability of a substance to retain moisture and oil, respectively [99].

Fruit by-product peels are often characterized by a high content of dietary fiber, which entails a high hygroscopicity. As a result, fruit peel flours tend to have a high water-holding capacity (WHC) and a relatively high oil-holding capacity (OHC), which contribute to moisture retention and directly affect the texture of food products. Higher water retention can be beneficial for applications such as burgers and meat analogs, in which it can act as a fat replacer and enhance juiciness and softness, thus increasing consumer acceptance [65,100]. However, when excessive, it can negatively affect the texture of foods such as breads and cakes, increasing crumb hardness and decreasing the height and specific volume [26,55], as well as in cookies, in which it increases spreadability and firmness and decreases height [58].

In the case of fruit albedos, the high fiber content and the considerable amount of pectin result in a high WHC, a good swelling capacity (i.e., the volume occupied by fiber in excess water), and good gelling properties, which improve its potential for application as a thickener or stabilizer in emulsions or to improve the texture of meat analogs [100]. Seed and kernel flours are also characterized by elevated WHC, in addition to the higher fat content and often higher OHC, being potential fat replacers in products such as burgers [65]. The OHC can also play a role in mouthfeel and flavor retention, which are crucial to improving consumer acceptance [20].

One of the main factors governing the techno-functional properties of fruit by-product flours is their composition, particularly in terms of fiber and protein content. Properties such as WHC and OHC are directly related to the total dietary fiber and the amount of insoluble fiber, respectively, whereas soluble fibers such as pectin and gums have a greater influence on gelling properties and proteins play a bigger role in emulsifying capacity [20,101]. Another defining factor is the processing of by-products into flours, especially regarding the drying parameters. Higher processing temperatures can affect the chemical structure of polysaccharides and promote lipid oxidation, thus impacting WHC and OHC, in addition to promoting protein denaturation and starch gelatinization, which reflect the emulsifying and gelling properties [20,102,103].

### 4.2. Applicability of Fruit By-Product Flours in Food Products

Despite their favorable nutritional properties, flours from fruit by-products pose a technological challenge when incorporated into food applications, such as bakery products, pasta, snacks, and beverages. Studies have shown that products with higher substitution levels of wheat flour with by-product flours tend to have lower consumer acceptance, particularly in terms of appearance, flavor, and texture [48,51,75,80,81,85]. One way in which fruit by-product flours negatively affect food products is by imparting a darker coloration, either due to a high content of polyphenolic compounds (e.g., anthocyanins from jaboticaba bark and peels), from their oxidation by polyphenol oxidase, or from a higher presence of sugars prone to undergoing non-enzymatic browning, as seen in products containing mango peel flours [53,56,57]. While some of these color changes can be prevented by blanching or autoclaving fruit by-products before drying and milling, substitution levels should be maintained below 10% for most applications to improve consumer acceptability.

The higher content of phenolic compounds in fruit by-products can also impart bitterness and astringency, which are considered deleterious in most applications and significantly reduce consumer acceptance [54]. Another way fruit byproducts can negatively impact flavor is by incorporating aroma compounds (e.g., limonene from guava and orange peels) that, while not deleterious by themselves, can hamper product acceptance by predominating over the expected aroma for a given product, as is the case for bread and other bakery products [77].

Another major impact that fruit by-product flours tend to have on food applications is on texture. The high fiber content of most by-products tends to improve water retention which, when allied with a good swelling capacity, highlights a potential use as a thickener, as a stabilizer for emulsions and foams, or to improve the juiciness of meat analogs [91]. However, in bakery products and pasta, higher water retention results in increased dough stickiness and hardness. Additionally, when the by-product flours replace wheat flour, especially above 10% substitution, they also hamper gluten formation, resulting in a weaker structure, a more closed crumb, and a lower specific volume [53,54,55].

All the flours included in this review demonstrated potential for use in baked foods and confectionery, particularly in cookies, cakes, and bread, even if higher levels of substitution (>10%) still pose a technological challenge. Additionally, banana peel, mango seed, orange peel, and jackfruit seed flours could be viable alternatives to wheat flour in gluten-free pasta products [54,74,97]. Passion fruit peel and banana peel flour also have potential applications in sweets, such as gingerbread and fruit snacks [62,96], while jackfruit seed flour, with its chocolate-like aroma, proved to be a good candidate in substituting cocoa powder while retaining the chocolate aroma [70,72].

### 4.3. Quality and Safety Control in Fruit By-Product Flour Processing

Despite the economic and environmental advantages of reincorporating fruit by-products as flours in modified or new foodstuffs, this approach requires the evaluation of suitable recycling and manufacturing processes to ensure the generation of non-injurious products that are adequate for human consumption or animal feed [9].

Regarding physicochemical quality, moisture and acidity are primordial parameters to evaluate the stability, microbiological safety, and hydrolytic rancidity of by-products and flours produced from them. Color determination and other physicochemical parameters, such as solubility and water and oil holding capacity (WHC and OHC), should define the potential application of fruit by-product flours. Additionally, the nutritional quality, in terms of protein, lipids, carbohydrates, and fibers, provides important pieces of information about the suitability of adding value to the by-product [9,60].

Microbiological quality is another attention point, since plant-based by-products, mainly from fruits, could develop undesirable microorganisms (such as *Salmonella*, *Listeria*, and *Escherichia coli*) if post-harvest conditions are inadequate, failing to fulfill the quality standards [9]. According to Brazilian regulation [104], *Salmonella* must be absent from all foodstuffs, and *E. coli* concentration must be below 10 CFU/g.

Finally, the risk of contamination of fruit by-products is extensive; therefore, the evaluation of contaminants, such as pesticides and toxins, should be carefully assessed to guarantee the quality and safety of the final products [9,105].

## 5. Conclusions

The processing of fruits that are essentially valued for their pulps results in substantial amounts of by-products that are typically discarded. This review highlighted the potential of adopting an upcycling approach to these by-products by turning them into flours that can be incorporated into various food formulations. This incorporation of by-product flours in food products not only improves their nutritional and functional properties, but also adds value to products that typically rely on refined flours, in addition to providing new alternatives for the use of different ingredients to reduce the waste generated throughout the fruit production chain.

Therefore, this review highlighted that by-product-enriched flours can contribute to reducing food waste, offering sustainable and nutritious alternatives for the food industry. However, further research is needed to explore sensory analysis techniques, higher substitution levels, and potential toxicity concerns. Industrial-scale studies will be crucial to confirm the viability and safety of using these flours in commercial food production.

## Data Availability

No new data were created or analyzed in this study. Data sharing is not applicable to this article.
